# Decoding Gene Expression Changes in Cerebral Tumors: Before and After Radiotherapy

**DOI:** 10.1002/med.22122

**Published:** 2025-06-01

**Authors:** Ahana Maitra, Morena Miciaccia, Manuela Mandorino, Domenico Armenise, Olga Maria Baldelli, Savina Ferorelli, Ludmila Papusha, Alexander Druy, Maria Grazia Perrone, Antonio Scilimati

**Affiliations:** ^1^ Research, Laboratory for Woman and Child Health, Department of Pharmacy ‐ Pharmaceutical Sciences University of Bari “Aldo Moro” Bari Italy; ^2^ Dmitry Rogachev National Medical Research Center of Pediatric Hematology Oncology and Immunology Moscow Russia

**Keywords:** cerebral tumor, emerging and future therapeutic strategies, gene expression profile, radiotherapy

## Abstract

Cerebral tumors, particularly in pediatric patients, pose a significant challenge in oncology. Radiotherapy is a crucial component of the multimodal treatment approach for these tumors. Understanding the molecular basis of these tumors, particularly their response to radiotherapy, is crucial for improving treatment outcomes and patient survival. Many cancer‐based studies have investigated gene expression patterns and gene signatures associated with radiotherapy. However, such studies are scarce in the field of pediatric cerebral tumors. Moreover, no studies have been conducted on the changes in gene expression profiles “before and after radiotherapy treatment in pediatric cerebral tumors,” especially in diffuse intrinsic pediatric glioma, actually classified as diffuse midline glioma. This review aims to explore the expression of gene profiles in cerebral tumors before and after radiotherapy, unraveling the molecular mechanisms underlying treatment response and potential biomarkers for prognosis and therapeutic targeting. By examining the current literature (years 2011–2023), we provide an overview of the present understanding of the gene expression changes associated with radiotherapy in intrinsic brain tumors. Insights from these studies suggest alterations in key signaling pathways, DNA repair mechanisms, and cell cycle regulation in response to radiotherapy. Our analysis highlighted potential genomic targets and the importance of identifying key genes and pathways involved in these responses to develop personalized treatment strategies and improve patient outcomes.

## 1 Introduction

Cerebral tumors are a significant health concern due to their complex heterogenous nature and potential impact on cognitive function and overall well‐being [1]. The importance of understanding brain tumors lies in their potential to cause severe neurological symptoms and their effect on a patient's quality of life. One vital aspect of brain tumors is their diverse range of types and subtypes, each with distinct characteristics and treatment implications. Advances in genomics, molecular profiling, and imaging technologies have enhanced our understanding of brain tumor biology, and it has been observed that among the various factors affecting brain tumor onset, development, and progression, epigenetic modifications, including DNA methylation, histone modifications, and noncoding RNAs, act as key contributors.

To date, radiation therapy plays a crucial role in the treatment of various tumors, including cerebral tumors, and the molecular effects of radiation on the gene expression profiles of cerebral tumors are not fully understood. Many gene expression profiling studies have been carried out in different tissues and cell types to identify specific genes and pathways involved in radiation‐induced cellular responses, including DNA damage recognition, repair mechanisms, cell cycle checkpoints, apoptosis, and inflammation in response to radiation [2, 3]. Genomic studies identified platelet‐derived growth factor receptor A (PDGFRA) gene mutations in high‐grade astrocytomas and glioblastomas (GBMs), particularly in the proneural subtype. PDGFRA mutations are common in GBMs and play a role in tumor progression. PDGFRA amplification is frequent in pediatric and adult GBMs, with a higher prevalence in de novo GBMs than in lower‐grade astrocytomas. This suggests its impact on tumor aggressiveness and treatment response. These findings highlight the importance of PDGFRA‐driven pathways in GBMs [4, 5], though further research is needed to understand their molecular characteristics better.

Several studies have been performed aimed at investigating gene signatures associated with radiotherapy in prostate cancer patients. For example, through a comprehensive analysis of gene expression data from prostate cancer patients who received radiotherapy, Kogionou et al. [6] identified a set of 6 genes (CCR7, FCGR2B, BTLA, CD6, CD3D, and CD3E) that were downregulated post‐radiotherapy compared to pre‐radiotherapy samples. Several similar studies established the potential of gene signatures as a predictive tool for assessing treatment outcomes and prognosis in prostate cancer patients undergoing radiotherapy [7, 8, 9].

Considerable research work has also been conducted to study epigenetic changes, such as DNA methylation, in breast cancer patients who received fractionated radiotherapy and develop gene signatures that can predict the radiosensitivity of breast cancer patients [10, 11]. Some studies focused on monitoring changes in gene expression patterns during adjuvant radiotherapy in breast cancer patients and used integrative analyses to identify radiation‐related genes and biomarkers associated with breast cancer [12, 13]. Similar studies have also been accomplished for rectal cancer [14, 15] and cervical cancer [16] patients.

Understanding the molecular basis of acute and persistent radiation responses is crucial for improving treatment outcomes and minimizing radiation‐induced side effects. By identifying key genes and pathways involved in these responses, it may be possible to develop targeted therapies that enhance tumor cell killing while sparing normal tissues. These gene signatures could potentially aid in selecting the most effective radiotherapy treatment options for individual cancer patients. However, such studies are yet to be conducted extensively for cerebral tumors, especially for pediatric brain tumors, as only a few have been found in the literature. Understanding how radiation influences the gene expression profiles can not only shed light on the underlying mechanisms of treatment response and resistance but also identify potential targets for intervention to sensitize tumors to radiation or overcome treatment resistance.

## 2 Literature Selection

Relevant studies and comprehensive reviews addressing gene expression profiles associated with radiotherapy in pediatric brain tumors were meticulously searched. However, we could not find any information about the changes in gene expression profiles before and after radiotherapy treatment in pediatric brain tumors. So, a narrative approach was adopted to create and analyze relevant studies and findings. The narrative review aimed to provide a cohesive narrative highlighting the current understanding of gene expression alterations associated with radiotherapy in intrinsic brain tumors, particularly on diffuse intrinsic pontine glioma (DIPG), also classified as diffuse midline gliomas (DMG). Henceforth, in this study, DIPG has been referred to as DMG.

We conducted a comprehensive literature search in PubMed to identify relevant studies published between 2011 and 2023. The search focused on three main areas: (i) the impact of radiotherapy treatment on gene expression profiles in patients with intrinsic brain tumors, (ii) gene expression profiles in brain tumors, with a specific focus on DMG, and (iii) differential gene expression and potential genomic targets for novel therapeutic approaches in DMG.

A literature search using the keywords “pediatric brain tumors,” “pre and postradiotherapy” and “gene expression profiles” returned zero (0) results. To ensure the comprehensiveness of the search, we expanded the search radius to include literature that is indirectly related to the mainstay of the study. Thus, we conducted multiple literature searches with additional keywords: “brain tumors,” “radiotherapy,” “diffuse midline glioma,” “therapeutic drugs affecting gene expression,” and “gene signatures,” which retrieved 181 studies. Studies that appeared in multiple searches were considered only once. Then, the publications were evaluated for relevance, and after careful investigation, 47 studies were deemed appropriate for this narrative review, primarily focusing on gliomas (intrinsic brain tumors), while 134 studies were excluded. Key findings and insights on gene expression alterations following radiotherapy in intrinsic brain tumors and future therapeutic strategies were extracted and synthesized to construct a coherent narrative.

This narrative review aims to address the scarcity of literature in the field and provide valuable insights into an emerging area of research. By consolidating and analyzing relevant studies from 2011 to 2023, we look forward to bridging the literature gap and contributing to the current understanding of gene expression profiles associated with intrinsic brain tumors and radiotherapy response.

## 3 Insights Into Gene Expression in Cerebral Tumors

### 3.1 Diffuse Midline Glioma (DIPG/DMG), Intrinsic Brain Tumors, and Gene Expression Profiles

Gene expression patterns have been studied for most intrinsic brain tumors. Overexpression of the ASPM gene has been identified as a potential molecular target in glioblastoma and medulloblastoma [17]. Similarly, in glioblastoma multiforme (GBM), changes in DNA repair and cell‐cycle gene expression were observed to occur during tumor development, and gene expression profiles of a 27 gene signature, protein‐coding genes (PCGs) and long noncoding RNAs (lncRNAs) were identified and analyzed [18, 19].

Clinical and molecular heterogeneity of medulloblastoma, a common malignant brain tumor in children, was unraveled by investigation of gene expression profiling. Northcott et al. [20] demonstrated that the four major medulloblastoma subtypes activate distinct genetic pathways, each contributing to tumor formation and progression. Group 1, the WNT group, is characterized by axon guidance signaling, which directs neural development but, when dysregulated, promotes uncontrolled cell proliferation. Additionally, WNT/β‐catenin signaling influences cell fate and proliferation; its activation in WNT tumors is associated with aggressive tumor behavior, making it a critical target for intervention. Alterations in O‐glycan biosynthesis, which affect protein modification and cell adhesion, may further contribute to tumor progression. Group 2, the SHH group, is essential for embryonic development but implicated in tumor growth when dysregulated. Furthermore, activating genes linked to human embryonic stem‐cell pluripotency suggests a role in sustaining tumorigenic potential. In group 3, phototransduction, glutamate receptor, and MAPK signaling indicate unique metabolic profiles and tumor‐promoting mechanisms, offering potential therapeutic targets. The group 4 subtype is influenced by p53 signaling, a crucial tumor suppressor pathway, alongside semaphorin signaling, which affects tumor behavior. These distinct pathways highlight the biological diversity of medulloblastoma and provide promising avenues for targeted therapies.

Analogously, ependymal tumors across all anatomic compartments were divided based on gene expression profiling, and subgroup‐specific pathways were identified [21]. Later, these findings formed the rationale for the DNA‐methylation‐based classification of brain tumors, as the methylation of cytidine nucleotides is a major and well‐investigated gene silencing mechanism. The biological and clinical diversity of choroid plexus carcinomas, rare primary brain tumors in children, was recently discovered by targeted gene expression profiling [22].

DMG is a highly malignant neoplasm that occurs primarily in children and young adults across the midline structures of the central nervous system, including the spinal cord, medulla oblongata, pons, and thalamus. The molecular hallmark of DMG is the loss of trimethylation at lysine 27 on histone H3 (H3K27me3) [23], a highly conserved epigenetic modification associated with transcriptional repression and heterochromatin formation. This loss is primarily driven by a recurrent missense mutation (Lys27Met; K27M) in one of the histone H3‐encoding genes, most commonly H3F3A (histone H3.3, ~75%), or less frequently H3C2, H3C3 (histone H3.1, up to 25%), and in rare cases, H3C14 (histone H3.2). A distinct subset of DMGs lacks mutations in the H3 gene family but exhibits overexpression of EZHIP (EZH inhibitory protein), which leads to a similar loss of H3K27me3. EZHIP functions as a natural antagonist of the polycomb repressive complex 2 (PRC2) by inhibiting the EZH1/EZH2 methyl‐transferase activity, thereby causing downregulation of histone H3 trimethylation on lysine 27 residue. This alternative mechanism of H3K27me3 depletion highlights the epigenetic dysregulation central to DMG pathogenesis [24, 25, 26, 27].

The major gene expression state in DMG cells is the overexpression of many genes due to the downregulation of PRC2, thus preventing the repression of genes associated with cancer development. Apart from cancer‐related genes, DMG cells alter the expression of genes involved in metabolic processes, such as methionine metabolism and the tricarboxylic acid cycle, as well as glucose and glutamine uptake [28]. Reciprocal to PRC2 inhibition, upregulation of SWI/SNF complex was noted in H3 K27M mutant DMG. Overexpression of *SMARCA2*, *SMARCA4*, and *PBRM1*, as well as key members of the forkhead family of transcription factors *FOXO1*, were considered therapeutic vulnerabilities of DMG [29, 30]. Another promising target for therapeutic interventions in DMG is the upregulated and phosphorylated (Tyr705) STAT3 protein [31].

Comparative analysis of gene expression profiles of DMG and healthy pons samples revealed upregulation of the *TGFB2* in tumors. Moreover, high levels of *TGFB2* mRNA predict extremely poor outcomes in DMG but not in other pediatric high‐grade gliomas. Interestingly, the expression of TGFB3 had the opposite effect, and its upregulation was associated with a slightly better prognosis [32].

A recent study describes diverse gene expression programs in different spatial compartments of DMG specimens. Cells from the core tumor compartment predominantly expressed genes associated with neuronal development, particularly those regulating oligodendrocyte precursor cells. On the other hand, cells from the vascular niche show enrichment in pathways related to reactive immune responses, hypoxia, and radial glia development, while those in the hypoxic niche exhibit strong activation of hypoxia and radial glia development signatures [33].

These distinct gene expression patterns (Table 1) can be used as biomarkers for investigating tumor behavior, particularly in response to radiotherapy‐induced epigenetic modifications. Understanding these signatures could provide critical insights into how radiotherapy reshapes the tumor epigenome and influences treatment outcomes. Similar to research in breast and prostate cancers, longitudinal pre‐ and post‐radiotherapy studies in cerebral cancers could uncover novel epigenetic regulatory mechanisms. Such insights would not only deepen our understanding of tumor plasticity and adaptation but also aid in refining personalized therapeutic strategies and improving the prognosis for patients with intrinsic brain tumors.

**Table 1:** Gene expression profiles associated with intrinsic brain tumor types.

Tumors	Gene Expression Profile	References
Glioblastoma and medulloblastoma	Overexpression of ASPM gene	[17]
Glioblastoma multiforme	Changes in DNA repair and cell‐cycle gene expression, identification of a 27‐gene signature, protein‐coding genes (PCGs), and long noncoding RNAs (lncRNAs)	[19]
Medulloblastoma	Activation of genes associated with − axon guidance signaling (WNT and SHH groups) − WNT/β‐catenin signaling − O‐glycan biosynthesis − human embryonic stem‐cell pluripotency − phototransduction pathway − glutamate receptor signaling − MAPK signaling − p53 signaling − semaphorin signaling − cAMP‐mediated signaling − G‐protein‐coupled receptor signaling	[20]
Diffuse midline glioma	Overexpression of the *EZHIP* gene due to − downregulation of PRC2, − upregulation of SWI/SNF complex − overexpression of SMARCA2, SMARCA4, PBRM1, FOXO1, and phosphorylated STAT3 protein	[24, 25, 26, 27, 28, 29, 30, 31]
Diffuse midline glioma	− Upregulation of TGFB2 (associated with a poor prognosis) − upregulation of TGFB3 (associated with a slightly better prognosis)	[32]

### 3.2 Gene Expression in Intrinsic Brain Tumors Upon Radiotherapy Treatment: Pre and Postradiotherapy Gene Expression

Among the earlier studies contributing to a better understanding of the molecular alterations as effects of radiotherapy on the gene profile expression of intrinsic brain tumors include the work by Joki et al. [34], which examined changes in gene expression in recurrent malignant glioma following radiotherapy using complementary DNA (cDNA) microarrays. Tumor samples from patients with recurrent malignant glioma who had undergone radiotherapy were collected, and their gene expression profiles were compared to those of untreated gliomas. The results revealed significant alterations in gene expression between recurrent malignant gliomas following radiotherapy and untreated gliomas. Significant alterations in gene expression were observed in recurrent tumors following radiation treatment, particularly in growth factor signaling pathways. Paracrine signaling factors, such as Vascular Endothelial Growth Factor (VEGF) and Platelet‐Derived Growth Factor Receptor β (PDGFRβ), exhibited reduced mRNA levels in recurrent tumors compared to primary tumors in three out of four patients. Similarly, autocrine signaling factors, including Epidermal Growth Factor Receptor (EGFR), Platelet‐Derived Growth Factor α (PDGFα), Platelet‐Derived Growth Factor A (PDGFA), and Basic Fibroblast Growth Factor (bFGF), also showed decreased expression in recurrent tumors. These findings highlight the genetic adaptations that occur in response to radiotherapy‐induced selective pressures, potentially altering tumor cell survival, proliferation, and microenvironment interactions. Understanding these gene expression changes may provide valuable insights into tumor recurrence and resistance mechanisms, offering potential molecular targets for future therapeutic strategies. These findings provided important insights into the genetic changes that occur in response to radiotherapy that could help identify potential targets for future therapeutic interventions.

Several studies have investigated the molecular mechanisms underlying the adaptive response of gliomas to radiation treatment. For example, Li et al. [35] identified a 5‐gene signature (HOXC10, LOC101928747, CYB561D2, RPL36A, and RPS4XP2) associated with radiotherapy response and prognosis through a comprehensive analysis of gene expression data from glioma patients who underwent radiotherapy. Another study focused on identifying genes aberrantly expressed in murine glioblastoma during radiotherapy using bioinformatic data mining approaches identified that Chemokine and IL‐6 signaling pathway‐associated genes were increased in the irradiated strains [36]. Raviraj et al. [37] reviewed the epigenetics of brain tumors, with a specific focus on the modulation of epigenetic changes during radiation therapy, and found that epigenetic modifications impact gene transcription, including Polycomb genes and V‐Myc avian myelocytomatosis viral oncogene (MYCN) in glioblastoma.

Matsko et al. [38] explored the link between the O‐6‐methylguanine‐DNA methyl‐transferase (MGMT) gene expression and the response to treatment in glioblastoma patients who received radiotherapy. Their findings revealed a remarkable decrease in glioblastoma size in patients with low levels of MGMT gene expression. MGMT is an enzyme involved in DNA repair, and its high expression has been associated with resistance to DNA alkylating agents, such as temozolomide (TMZ), concomitantly used with radiotherapy [39]. Notably, MGMT promoter methylation correlated with a positive response to TMZ in glioblastoma patients [40, 41] and showed prognostic value even in those glioblastoma patients who did not receive TMZ with radiotherapy [42]. Despite TMZ use, no studies have demonstrated a clear role of temozolomide in the setting of DIPG, and it is largely an extrapolation of glioblastoma therapy. Studies support a high expression of MGMT in H3 K27‐altered DMGs, contributing to temozolomide resistance in DIPG. Despite advances in medical care and preclinical and clinical studies, the median overall survival of DIPGs is 9–15 months, a figure that has remained unchanged for decades. Lower MGMT expression has been associated with favorable treatment outcomes for glioblastoma patients [38], which could also be regarded as the cause and not the effect of irradiation. However, all studies indicate that MGMT gene expression levels could serve as a predictive biomarker for treatment response in glioblastoma.

In a recent study, Walker et al. [43] investigated the molecular mechanisms underlying the adaptive response of glioma cells to radiation treatment. They identified a key protein complex called PTEFb, whose inhibition leads to the disruption of chromatin reorganization, resulting in diminished transcriptional induction and impaired DNA damage repair and cell cycle regulation. The study showed that exposure to radiation leads to rapid reorganization of active chromatin, enabling PTEFb‐mediated transcriptional induction within a few hours.

Akkari et al. [44] explored how glioma macrophage population**s** change dynamically after radiotherapy and suggested that inhibition of the colony‐stimulating factor‐1 receptor (CSF‐1R) can be used as a therapeutic strategy to overcome resistance and improve survival in preclinical models. They also identified specific gene expression patterns after irradiation in murine gliomas and confirmed altered gene and protein expression in recurrent human glioblastoma. Cifarelli et al. [45] studied molecular characteristics underlying the diverse radiation responses through molecular profiling and observed distinct gene expression patterns and molecular signatures, which provided insights into the biological processes and pathways involved in mesenchymal glioblastoma and its response to radiation therapy.

Another study [46] focused on the development and validation of a gene expression signature that can predict prognosis in lower‐grade glioma (LGG) patients who have undergone surgery and adjuvant radiotherapy. Analysis of gene expression data, recorded in The Cancer Genome Atlas (TCGA), related to a cohort of 289 LGG patients identified 5 genes (MAP3K15, MAPK10, CCL3, CCL4, and ADAMTS1) significantly associated with patient outcomes, specifically the overall survival.

These results suggest that these prognostic gene expression signatures have potential clinical utility in guiding treatment decisions and predicting outcomes for patients with intrinsic brain tumors who undergo surgery and adjuvant radiotherapy (Table 2). They also provide a valuable tool for identifying patients requiring more aggressive therapeutic approaches or closer surveillance. Furthermore, insights gained from gene expression profiling studies can contribute to the identification of novel molecular targets for radiosensitization, allowing for more effective radiation treatment regimens. Altogether, these findings may lead to future ways of developing personalized treatment strategies and targeted therapies to improve outcomes for patients with intrinsic brain tumors.

**Table 2:** Genes associated with radiotherapy response in intrinsic brain tumors.

Gene	Association with radiotherapy	References
Vascular endothelial growth factor (VEGF)	Reduction in mRNA levels in recurrent malignant gliomas postradiotherapy	[34]
Platelet‐derived growth factor receptor β (PDGFRβ)	Reduction in mRNA levels in recurrent malignant gliomas postradiotherapy	[34]
Epidermal growth factor receptor (EGFR)	Decreased mRNA levels in recurrent malignant gliomas postradiotherapy	[34]
Platelet‐derived growth factor α (PDGFα)	Decreased mRNA levels in recurrent malignant gliomas postradiotherapy	[34]
Platelet‐derived growth factor A (PDGF A)	Decreased mRNA levels in recurrent malignant gliomas postradiotherapy	[34]
Basic fibroblast growth factor (bFGF)	Decreased mRNA levels in recurrent malignant gliomas postradiotherapy	[34]
HOXC10 LOC101928747 CYB561D2 RPL36A RPS4XP2	Identified as biomarkers associated with radiotherapy response and prognosis in glioma patients	[35]
Chemokine and IL‐6 signaling pathway‐associated genes	Increased expression during radiotherapy	[36]
O‐6‐methylguanine‐DNA methyl‐transferase (MGMT)	Low levels associated with reduction in glioblastoma size postradiotherapy	[38]
PTEFb	Implicated in chromatin reorganization and DNA damage repair postradiotherapy	[43]
Colony‐stimulating factor‐1 receptor (CSF‐1R)	Dynamic changes postradiotherapy, potential therapeutic target	[44]
MAP3K15 MAPK10 CCL3 CCL4 ADAMTS1	Associated with overall survival in lower‐grade glioma patients postradiotherapy	[46]

### 3.3 Molecular Insights and Emerging Therapeutic Strategies for Diffuse Midline Glioma

DMG is a highly lethal disease, with most patients surviving less than 1 year after diagnosis and fractioned radiotherapy being the first‐line treatment. Despite various treatment attempts, overall survival has not improved significantly [1]. Given the pressing need to enhance survival rates, there is a growing interest in better comprehending the molecular characteristics of DMG and facilitating personalized treatment approaches, as traditional radiological imaging alone has not been sufficient for substantial progress.

To understand the complex genetics of pediatric brain stem gliomas (BSGs), multiple studies have been conducted in the last two decades. One of the important primary studies in this direction was the investigation of ERBB1 amplification and overexpression in BSGs and their relationship with TP53 expression and mutations [47]. ERBB1 gene expression significantly increased with higher‐grade tumors, while TP53 abnormalities were grade‐independent, suggesting that ERBB1 signaling could be a potential therapeutic target for childhood BSGs. In another study, Li et al. [48] assessed the presence of EGFRvIII expression in pediatric DMG samples and found that EGFRvIII was expressed in a significant portion of the samples, suggesting that it may be a potential target for treatment in these often fatal pediatric tumors.

**Figure 1:**
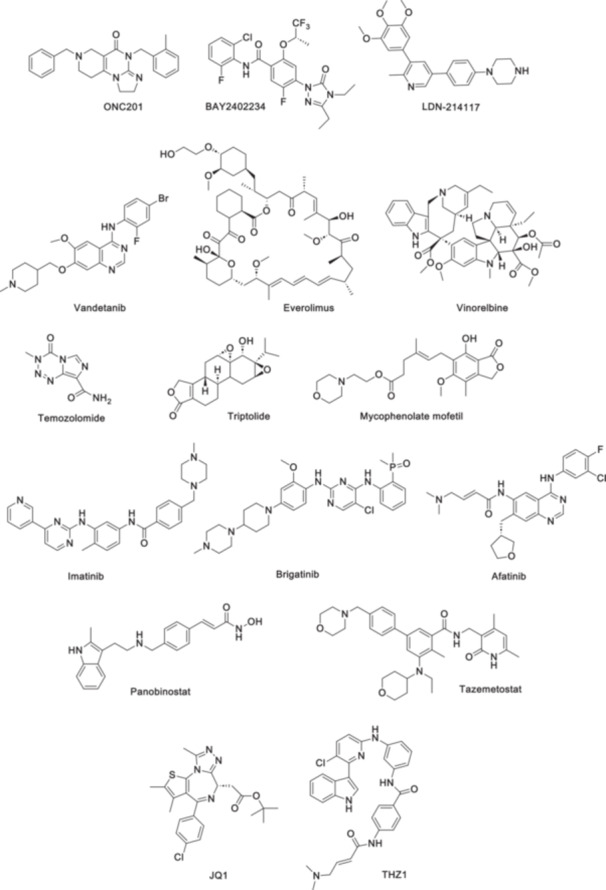
Chemical structure of some drugs and Inhibitors for the treatment of diffuse midline glioma (DMG).

ONC201 (Figure 1), a drug with properties as a DRD2 antagonist and mitochondrial ClpP activators [1], has shown promise in early responses for patients with DMG harboring the H3 K27M mutation, a condition for which there are limited treatment options beyond radiation therapy [49]. This study also uncovered a potential resistance mechanism involving an EGFR/FOXG1‐driven gene regulatory network, suggesting future combination therapy possibilities by targeting EGFR.

Researchers also conducted a genome‐wide CRISPR screen by targeting the rate‐limiting enzyme DHODH with the clinical‐stage inhibitor BAY2402234 (Figure 1), which reduced uridine‐5’‐phosphate (UMP) pools, induced DNA damage, and triggered apoptosis, ultimately extending the survival of mice with intracranial DMG xenografts [50]. By exploiting this metabolic vulnerability, this study highlights BAY2402234 as a promising therapeutic option for DMGs.

Somatic mutations in ACVR1 are common in DMG [51], and treatment with ALK2 inhibitors (ALK2i) has shown promising results in reducing cell viability and prolonging survival in animal models. However, as standalone treatments, these inhibitors could not achieve a complete antitumor response. A novel artificial intelligence study identified a combination therapy involving vandetanib and everolimus (Figure 1) as a potential treatment approach [52]. The tyrosine kinase inhibitor vandetanib, which inhibits VEGFR/RET/EGFR, targets ACVR1 and reduces DMG cell viability In Vitro. When combined with the mTOR/ABC transporter inhibitor everolimus, the survival results were extended, and tumor burden in a DMG xenograft model was reduced.

In another recent study [53], a single‐center experience was conducted in treating patients with DMG by comparing targeted therapies with nimotuzumab/vinorelbine or temozolomide (Figure 1) (affected mTOR/p‐mTOR pathway and BRAF V600E) with standard care. The first‐line treatment included radiotherapy and specific drugs based on the molecular profile of the tumor. The overall survival (OS) rate was significantly higher in patients who received personalized, targeted therapies than those treated with standard care. Notably, everolimus was identified as the treatment associated with the best overall survival in this study.

Through a systematic computational approach using publicly available databases and gene signatures from DMG patients and cancer cell lines, Zhao et al. [54] investigated therapeutic agents capable of reversing the DMG gene signature to resemble normal tissue. They identified two drugs, triptolide and mycophenolate mofetil (MMF), demonstrating significant inhibition of DMG cell viability. MMF treatment also inhibited tumor growth in In Vivo models, suggesting the potential of these clinically available drugs for treating DMG.

Another interesting approach was discussed in the extensive review by Nazh et al. [55] regarding the application of anti‐GD2 Chimeric Antigen Receptor T cells (CAR‐T cells) for neuroblastoma and gliomas. Engineered CAR‐T cells, designed to recognize disialoganglioside GD2 (a disialoganglioside belonging to b‐series ganglioside), exhibit promising durability and potency, with the added capability to penetrate the blood‐brain barrier. Ongoing clinical trials focus on pediatric and adult patients with high‐grade GD2‐expressing gliomas [56, 57].

Srikanthan et al. [58] discuss the landscape of clinical trials and targeted therapies for DMG, stating that approximately 250 clinical trials have been initiated targeting various biological pathways, with PDGFRA and EGFR being among the frequently amplified genes. However, therapies targeting PDGFRA, such as imatinib and dasatinib (Figure 1), have shown limited effectiveness [59]. Clinical trials exploring anti‐EGFR drugs (nimotuzumab, gefitinib, and erlotinib) have demonstrated some benefit in specific subsets of DMG patients [60, 61, 62]. JMJD3 inhibitors, including panobinostat and GSK‐4, targeting histone deacetylase and demethylase, have moved into clinical trials, showing promising results [63, 64]. Chromatin remodelers, such as EZH2, have been targeted, with promising results with tazemetostat (Figure 1) [65]. Transcriptional regulators like BET family proteins (JQ1) and CDK7 inhibitors (THZ1) have also been investigated for their therapeutic potential in DMG [66, 67].

To sum up, the current research landscape in DMG emphasizes the urgent need for effective therapeutic strategies to improve patient outcomes. Despite the grim prognosis of DMG, recent studies have revealed potential avenues for personalized treatment approaches (Table 3). Notably, ONC201 has exhibited early promise in targeting DIPG/DMG harboring the H3 K27M mutation, while genome‐wide CRISPR screens have identified metabolic vulnerabilities exploitable with inhibitors like BAY2402234. Additionally, combination therapies, such as vandetanib and everolimus, have shown efficacy in preclinical models, emphasizing the importance of synergistic approaches. Furthermore, personalized, targeted therapies guided by molecular profiling have demonstrated superior outcomes compared to standard care, with everolimus emerging as an up‐and‐coming agent. Exciting advancements in immunotherapy, including anti‐GD2 Chimeric Antigen Receptor T cells (CAR‐T cells), also offer potential avenues for effective treatment. However, challenges remain, especially in overcoming resistance mechanisms and optimizing therapeutic regimens.

**Table 3:** Drugs and inhibitors (Figure 1) for the treatment of diffuse midline glioma (DMG) and their molecular effects.

Drug/Inhibitor	Molecular effects	References
ONC201	DRD2 antagonist, mitochondrial protease ClpP inducer/activator	[1, 49]
BAY2402234	Reduces UMP pools, induces DNA damage, triggers apoptosis	[50]
ALK2 inhibitors (ALK2i) (e.g., LDN‐214117)	Reduces cell viability and prolongs survival in animal models	[51]
Vandetanib and everolimus combination therapy	Inhibits VEGFR/RET/EGFR, reduces DMG cell viability, reduces tumor burden	[52]
Nimotuzumab/vinorelbine or temozolomide	Affects the mTOR/p‐mTOR pathway and BRAF V600E, improves overall survival	[53]
Triptolide and mycophenolate mofetil (MMF)	Inhibits DIPG/DMG cell viability and tumor growth in In Vivo models	[54]
Anti‐GD2 Chimeric Antigen Receptor T cells (CAR‐T cells)	Recognizes disialoganglioside GD2, penetrates the blood‐brain barrier	[55, 56, 57]
PDGFRA and EGFR inhibitors (e.g., imatinib for PDGFRA; brigatinib and afatinib as EGFR reversible and irreversible inhibitors, respectively)	Target frequently amplified genes	[58, 59, 60, 61, 62]
JMJD3 inhibitors (panobinostat, GSK‐4)	Target histone deacetylase and demethylase	[58, 63, 64]
EZH2 inhibitors (tazemetostat)	Target chromatin remodelers	[58, 65]
BET family protein inhibitors (JQ1)	Target transcriptional regulators	[58, 66]
CDK7 inhibitors (THZ1)	Target transcriptional regulators	[58, 67]

## 4 Perspectives

Currently, radiotherapy is the “gold standard” treatment used and very often combined with chemotherapy. Radiotherapy is a palliative treatment as it alleviates symptoms. Chemotherapeutic drugs do not lead to results as they are mostly unable to cross the blood‐brain barrier (BBB). BBB plays an important role in regulating central nervous system (CNS) homeostasis, and in this case, it represents an obstacle for many low lipophilic drugs to reach it (Lipinski's rule of five). Preliminary studies were performed using Focused Ultrasound (FUS) to open BBB temporarily, allow a milder use of radiotherapy, and facilitate the passage of BBB from drugs. There are some ongoing investigations on patient‐derived xenograft DMG mouse model (Olaparib) [68, 69] and clinical trials in which DMG patients H3 K27‐altered are treated with FUS and ONC201 (NCT), doxorubicin (NCT05123534, NCT05615623, NCT05630209) [70], etoposide (NCT05762419), panobinostat (NCT04804709). These studies have not yet been concluded, but the corresponding interim results are available. We hope that in the near future, due to the lack of data, the clinical protocol used to manage DMG patients should include the possibility of performing a biopsy even after post‐RT treatment to detect changes in the gene expression profile compared to profile detected in the biopsy performed before administration of RT, to guide clinicians in choosing the appropriate personalized multi‐targeted treatment.

## 5 Conclusion

In conclusion, gene expression profiling studies have provided valuable insights into the molecular mechanisms underlying acute and persistent responses to radiation. Such studies have important implications for optimizing radiation therapy, tailoring treatment strategies, and improving patient outcomes. By integrating knowledge of epigenetic alterations and their response to radiation, we can pave the way for personalized treatment approaches and improve outcomes for patients with pediatric brain tumors, especially in the case of DIPG/DMG [66, 67, 71, 72, 73, 74]. We hope to raise awareness worldwide so that extensive comparative studies are conducted on the gene expression profiles before and after radiotherapy. Understanding the gene expression changes occurring during irradiation will pave the way for better prognosis and personalized approaches to cancer treatment.

In conclusion, the intricate landscape of cerebral tumors, particularly pediatric brain tumors like DIPG, presents a complex challenge in terms of diagnosis and treatment. Despite significant advancements in genomics, molecular profiling, and imaging technologies, much remains to be elucidated regarding the molecular effects of radiotherapy on gene expression profiles in these tumors. While studies in other cancer types have provided valuable insights into the genetic alterations induced by radiotherapy and their implications for treatment outcomes, research in pediatric brain tumors, especially DIPG, remains limited.

## Author Contributions

Conceptualization: Antonio Scilimati, Maria Grazia Perrone, and Ahana Maitra. Methodology: Antonio Scilimati, Ludmila Papusha, Alexander Druy, and Ahana Maitra. Writing – original draft preparation: Ahana Maitra and Antonio Scilimati. Writing – review and editing: Ahana Maitra, Manuela Mandorino, Domenico Armenise, Olga Maria Baldelli, Morena Miciaccia, Savina Ferorelli, Ludmila Papusha, Alexander Druy, and Maria Grazia Perrone. Supervision: Maria Grazia Perrone and Antonio Scilimati. All authors have read and agreed to the published version of the manuscript.

## Ethics Statement

The authors have nothing to report.

## Conflicts of Interest

The authors declare no conflicts of interest.

## Data Availability

Data sharing does not apply to this article, as no datasets were generated or analyzed during the current study.
